# Bladder Amyloidosis With Pulmonary Lesions: A Case of Spontaneous Remission After Transurethral Resection

**DOI:** 10.1002/iju5.70123

**Published:** 2025-12-28

**Authors:** Suguru Ito, Takuma Suzuki, Ryo Sato, Kunihiko Maeda, Shinta Suenaga, Hidenori Kanno, Sadanobu Sato, Shigemitsu Horie, Sei Naito, Norihiko Tsuchiya

**Affiliations:** ^1^ Department of Urology Okitama General Hospital Kawanishi, Higashiokitama Yamagata Japan; ^2^ Department of Urology Yamagata University Faculty of Medicine Yamagata City Yamagata Japan; ^3^ Department of Hematology Yamagata University Faculty of Medicine Yamagata City Yamagata Japan; ^4^ Department of Hematology Okitama General Hospital Kawanishi, Higashiokitama Yamagata Japan; ^5^ Department of Pathology Okitama General Hospital Kawanishi, Higashiokitama Yamagata Japan

**Keywords:** AL (λ) amyloidosis, bladder amyloidosis, plasma cell, spontaneous remission, transurethral resection

## Abstract

**Introduction:**

Bladder amyloidosis is a rare condition characterized by amyloid fibril deposition in the bladder, often mimicking bladder cancer. Diagnosis requires histological analysis of the examination, and treatment typically involves transurethral resection.

**Case Presentation:**

An 80‐year‐old male presented with gross hematuria. Computed tomography suggested bladder cancer with pulmonary metastases. However, due to the extent of the bladder tumor, complete resection was not feasible, and only a limited transurethral resection was performed for diagnostic purposes. Pathological examination revealed bladder amyloid λ amyloidosis. Remarkably, five months post‐surgery, both bladder and pulmonary lesions resolved spontaneously. No recurrence was observed during six years of follow‐up.

**Conclusions:**

This case highlights a rare instance of spontaneous remission in bladder amyloidosis. However, such occurrences were exceedingly uncommon, and systemic amyloidosis can progress to a life‐threatening condition. Therefore, careful follow‐up, surgical removal, or systemic therapy is essential for bladder amyloidosis.

## Keynote Message

- Bladder amyloidosis is a rare condition that can mimic bladder cancer.
- Spontaneous remission of bladder amyloidosis is extremely rare as demonstrated in this case.
- Systemic amyloidosis can be life‐threatening, requiring careful follow‐up, surgical intervention, or systemic therapy.

## 1 Introduction

Amyloidosis is a group of disorders characterized by the deposition of misfolded proteins in various organs, leading to tissue damage and organ dysfunction [1]. Amyloidosis is broadly classified into systemic and localized forms. Systemic amyloidosis frequently affects, the heart, kidneys, and nervous system, and can progress to a life‐threatening condition. In contrast, localized amyloidosis is confined to a single organ and generally has a relatively favorable prognosis. Recent advancements in research have improved the understanding of amyloidosis pathobiology, leading to progress in diagnosis and treatment [2]. Amyloidosis can be classified based on the types of amyloid deposit, which are identified through immunohistochemistry. The main types include Amyloid Light‐chain (AL), Amyloid Associated (AA), Amyloid Transthyretin (ATTR), and Amyloid Beta‐2 Microglobulin (Aβ2M), with treatment strategies varying depending on the amyloid type [3]. Each subtype has distinct pathogenic mechanisms—AL is associated with monoclonal plasma cells, AA with chronic inflammation, ATTR with transthyretin gene mutations or aging, and Aβ2M with long‐term dialysis. Bladder amyloidosis is a rare manifestation of amyloidosis and can macroscopically mimic bladder tumors, requiring careful differential diagnosis. Herein, we report a unique case of bladder amyloidosis that underwent spontaneous remission and discuss its clinical implications in the context of the relevant literature.

## 2 Case Report

**FIGURE 1:**
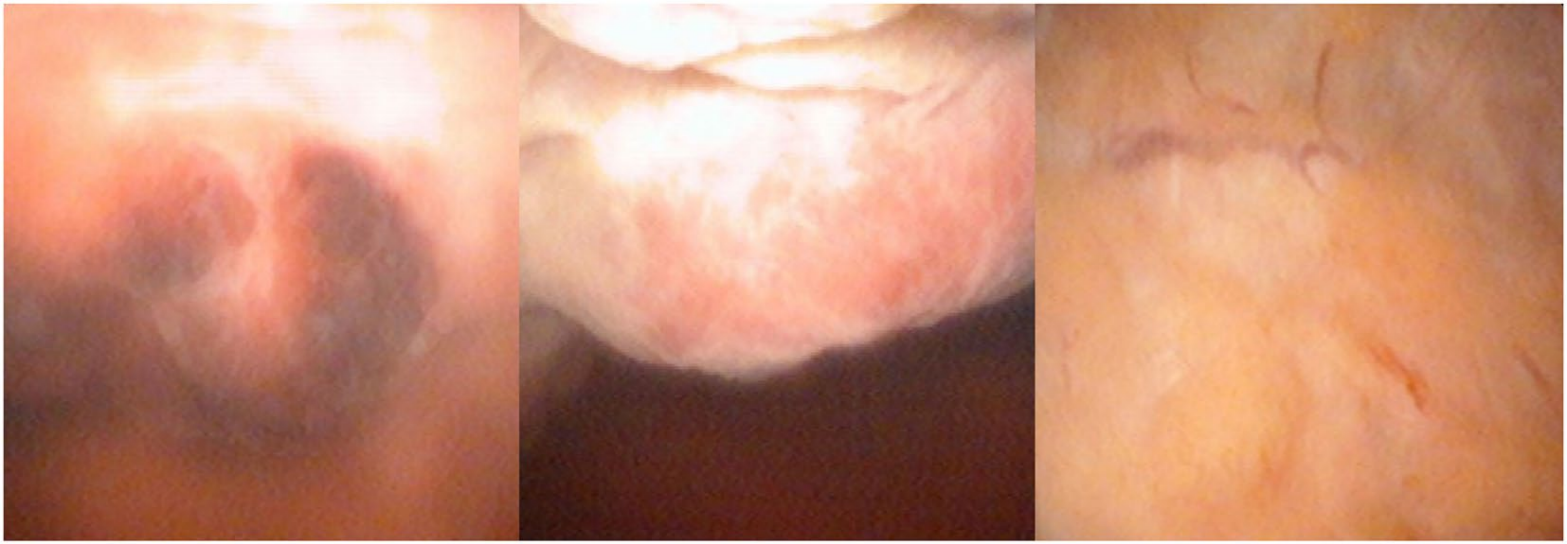
Cystoscopic findings at initial examination: multiple broad‐based nodular tumors.

**FIGURE 2:**
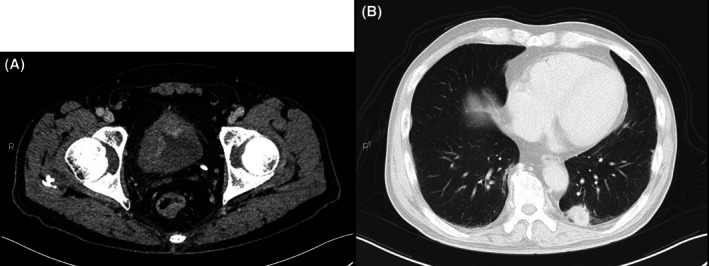
CT findings. (A) Nodule in the left pulmonary. (B) Multiple bladder tumors.

An 80‐year‐old man presented to his primary care physician with gross hematuria and was referred to our department for further evaluation. He had a medical history of hypertension, dyslipidemia, and hyperuricemia. Urinalysis revealed hematuria and proteinuria (2+), without pyuria or bacteriuria. Laboratory tests showed a white blood cell count of 11 300/μL and C‐reactive protein of 1.25 mg/dL, indicating mild inflammation, but no other remarkable abnormalities. Urine cytology was suspicious for malignancy. Cystoscopy revealed multiple nodular, broad‐based tumors located on the posterior wall, bladder dome, and anterior wall (Figure 1). Computed tomography (CT) suggested extra‐vesical invasive bladder cancer, with a 32‐mm pulmonary metastasis (Figure 2).

**FIGURE 3:**
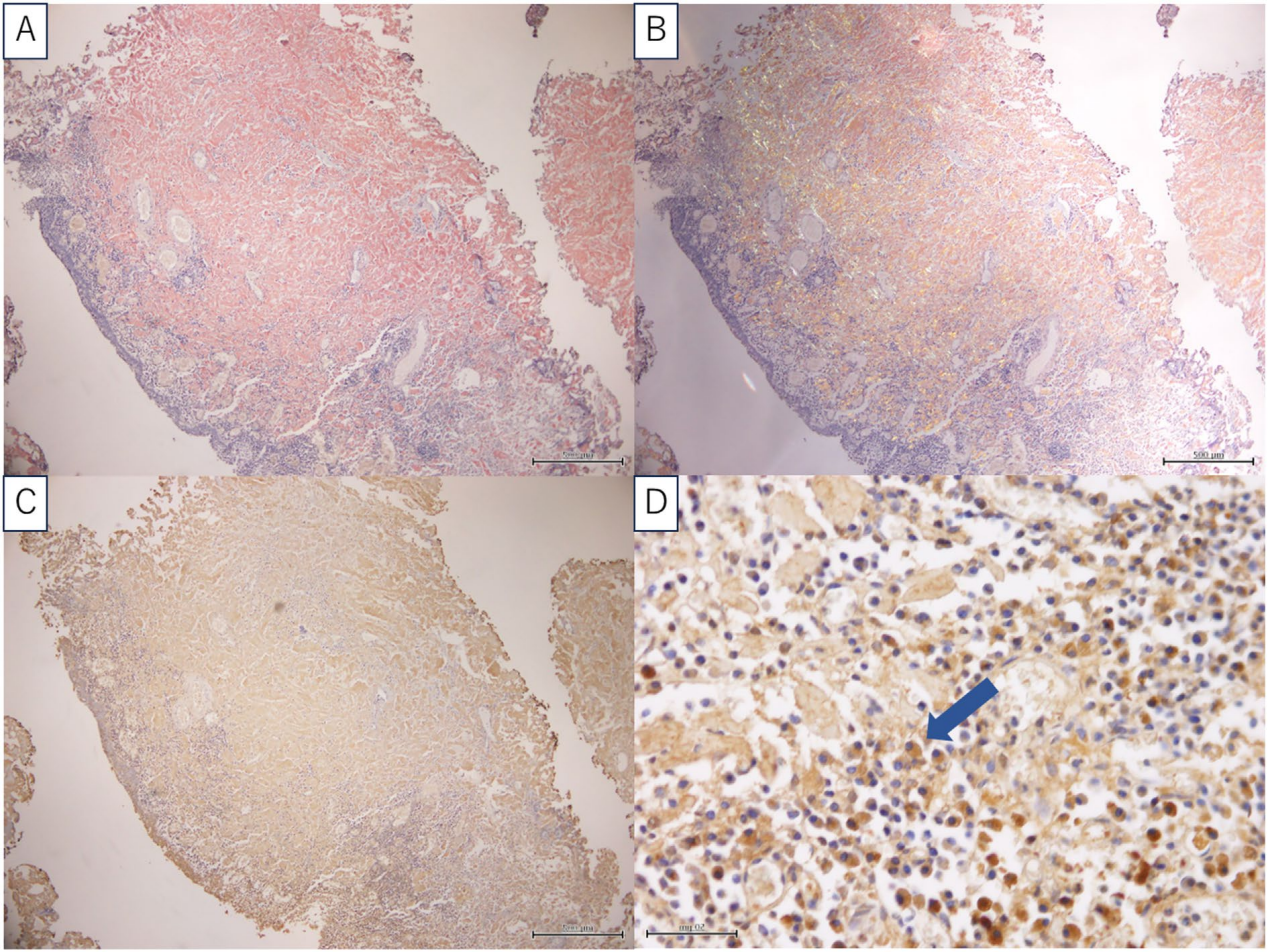
Pathological findings. (A) Bladder tissue stained with Congo red, highlighting amyloid deposits. (B) Under polarized light microscopy, amyloid deposition exhibits characteristic apple‐green birefringence. (C) Immunohistochemical staining reveals positive reactivity for lambda‐type amyloid. (D) High‐magnification observation demonstrates scattered plasma cells producing lambda‐type amyloid.

He underwent transurethral resection (TUR), with residual tissue remaining due to extravesical disease. Pathological examination revealed Congo red positive findings (Figure 3). Immunohistochemistry was positive for amyloid λ. Based on these findings, a diagnosis of bladder AL λ amyloidosis was made.

The pulmonary nodule identified on CT was suspected to be related to amyloidosis, and a bronchoscopy was performed. However, no nodule was identified, and laboratory testing, including serum proteins (IgG, IgM, IgA, β2‐microglobulin) and serum free light chains (κ, λ, κ/λ ratio), revealed no significant abnormalities.

**FIGURE 4:**
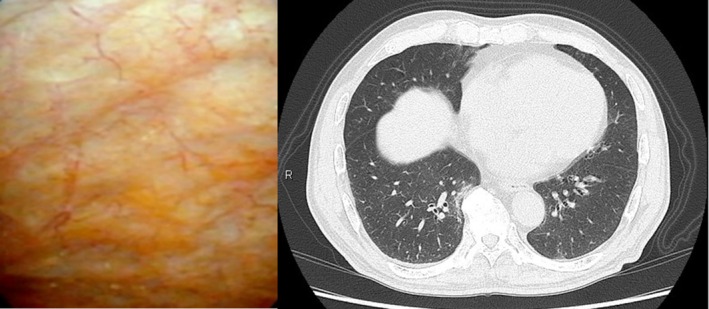
Cystoscopy and chest CT at 5 months post‐surgery. The tumors have disappeared.

Five months after surgery, follow‐up CT and cystoscopy showed the pulmonary and residual bladder lesions had completely disappeared (Figure 4). Based on the absence of cardiac and gastrointestinal involvement as well as negative blood test findings, the disease was considered localized, and the patient has been kept under regular follow‐up without recurrence for six years.

## 3 Discussion

AL amyloidosis consists of λ and κ types, both produced by B cells and plasma cells. AL amyloidosis can be either localized or systemic, with the λ more often systemic and the κ more frequently localized. Systemic AL amyloidosis is treated with chemotherapy or autologous hematopoietic stem cell transplantation [4], while AA is managed by controlling the underlying disease or using biological agents [5]. For ATTR amyloidosis, transthyretin‐stabilizing drugs or synthetic inhibitors are utilized [6]. Despite evolving drug therapies, surgical resection remains the first‐line treatment for localized amyloidosis, where systemic abnormal clones are typically absent.

Bladder amyloidosis is rare, primarily presenting with hematuria and lower urinary tract symptoms [7]. Cystoscopy findings vary, including solitary or multiple lesions, nodular or papillary tumors complicating differential diagnosis with bladder cancer. Diagnosis requires TUR and pathological examination [8], confirmed by Congo red staining exhibiting apple‐green birefringence under polarized light microscopy. Additionally, cancer comorbidity must be considered, as urothelial carcinoma has been reported in 48% of bladder amyloidosis cases [9]. In an analysis of 17 cases, ATTR amyloidosis was identified in 10 cases, AL amyloidosis in 3, AA amyloidosis in 1, and three cases were unclassifiable [9].

For localized bladder amyloidosis, TUR is the preferred treatment, with a success rate of 89.1% when completely resectable. In 20 completely unresectable cases due to diffuse multifocal amyloidosis of the bladder, the addition of colchicine and intravesical treatment with dimethyl sulfoxide was attempted. In cases unresponsive to these treatments, or in patients presenting with massive hematuria or severe irritative bladder symptoms, radical cystectomy or partial cystectomy has been reported as a treatment option [10]. There is no established protocol for follow‐up; however, some reports recommend cystoscopy at 3 months postoperatively, and then at 1 or 3 years, with further evaluation advised if gross hematuria or other urinary symptoms develop [11]. Systemic amyloidosis, necessitates tailored systemic therapy along with amyloid type [10]. Recurrence is common, occurring in 35.3% of cases, with a mean time to first recurrence of 20 months [12].

The spontaneous resolution observed in this case is exceptional and aligns with the limited reports documenting similar occurrences in localized amyloidosis. Spontaneous remission of bladder amyloidosis is exceedingly rare, with only one case reported by Jia et al. [13], although its subtype was unspecified. Spontaneous remission has been observed in other forms, including bronchial amyloidosis [14], renal amyloidosis [15] and pulmonary amyloidosis [16], although prior reports generally did not discuss possible mechanisms, probably reflecting the fact that the detailed classification and pathophysiology of amyloidosis have only been clarified in recent years.

In this case, the pulmonary lesion disappeared before examination, preventing definitive amyloidosis confirmation. Additionally, comprehensive investigations, including blood tests, did not indicate systemic involvement, leaving uncertainty regarding systemic versus localized disease. Given the rarity of spontaneous remission and the high systemic amyloidosis rate λ type, careful follow‐up is warranted in this case. The mechanisms underlying the spontaneous remission of amyloidosis remain unclear.

Our hypothesis is that TUR effectively removes plasma cells, reducing amyloid protein production and subsequent deposition, potentially contributing to disease resolution. While classification and treatment strategies for amyloidosis have advanced, many aspects remain unresolved. In systemic cases, therapy differs by immunoglobulin subtype, necessitating accurate subtyping. In Japan, a national pathology consultation working group has been established to support the diagnosis of amyloidosis, assisting clinicians in identifying the specific subtype and facilitating appropriate treatment decisions.

In bladder amyloidosis, localized lesions are common, making local resection effective. However, systemic amyloidosis requires distinct therapeutic approaches, necessitating thorough evaluation. Although spontaneous remission, as seen in this case, is possible, it remains rare. Even after successful treatment, careful follow‐up is essential to monitor for recurrence or systemic progression.

## Ethics Statement

The research protocol was approved by the Institutional Review Board of Okitama General Hospital. The approval number is 399.

## Consent

Informed consent was obtained.

## Conflicts of Interest

Sei Naito and Norihiko Tsuchiya are the editorial board members of the International Journal of Urology and the co‐authors of this article. To minimize bias, they were excluded from all editorial decision‐making related to the acceptance of this article for publication.

## Data Availability

Data sharing not applicable to this article as no datasets were generated or analyzed during the current study.

## References

[1] I. Vaxman and M. Gertz , “When to Suspect a Diagnosis of Amyloidosis,” Acta Haematologica 143 (2020): 304–311, 10.1159/000506617. 32340017

[2] K. R. Baker and L. Rice , “The Amyloidoses: Clinical Features, Diagnosis and Treatment,” Methodist DeBakey Cardiovascular Journal 8 (2012): 3, 10.14797/mdcj-8-3-3. PMC3487569 23227278

[3] B. P. Hazenberg , “Amyloidosis,” Rheumatic Disease Clinics of North America 39 (2013): 323–345, 10.1016/j.rdc.2013.02.012. 23597967

[4] M. Locke and M. Nieto , “AL Amyloidosis: Current Treatment and Outcomes,” Advances in Hematology 2025 (2025): 7280805, 10.1155/ah/7280805. 40226119 PMC11991823

[5] S. Mirioglu , O. Uludag , O. Hurdogan , et al., “AA Amyloidosis: A Contemporary View,” Current Rheumatology Reports 26 (2024): 248–259, 10.1007/s11926-024-01147-8. 38568326 PMC11219434

[6] T. M. Capovilla , A. Lalario , M. Rossi , et al., “Tafamidis in the Treatment of ATTR‐Related Cardiomyopathy: Indications and Grey Zones,” Heart Failure Clinics 20 (2024): 333–341, 10.1016/j.hfc.2024.03.007. 38844304

[7] R. Fitzpatrick , N. R. Paterson , E. Belanger , A. McCurdy , and J. Watterson , “Primary Amyloidosis of the Bladder; a Mimicker of Bladder Cancer,” Canadian Journal of Urology 24 (2017): 8868–8870. 28646945

[8] C. Ng , W. H. Huang , H. C. Huang , L. J. Wang , and S. Y. Lee , “Localized Bladder Amyloidosis Mimicking Bladder Carcinoma,” Kidney International 85 (2014): 1245, 10.1038/ki.2013.312. 24786889

[9] D. Sirohi , J. Gandhi , M. Amin , M. B. Amin , and D. J. Luthringer , “Amyloidosis of the Bladder and Association With Urothelial Carcinoma: Report of 29 Cases,” Human Pathology 93 (2019): 48–53, 10.1016/j.humpath.2019.08.011. 31425694

[10] N. Pyrgidis , I. Mykoniatis , V. F. Pegios , et al., “Amyloidosis of the Urinary Bladder: A Systematic Review and a Proposed Management Algorithm,” Urology 156 (2021): e12–e19, 10.1016/j.urology.2021.07.013. 34314752

[11] M. Wilkinson , D. M. Fanning , and H. Flood , “Primary Bladder Amyloidosis,” BMJ Case Reports 2011 (2011): bcr0520114211, 10.1136/bcr.05.2011.4211. PMC3143334 22689606

[12] A. Hosseini , A. Ploumidis , C. Adding , et al., “Radical Surgery for Treatment of Primary Localized Bladder Amyloidosis: Could Prostate‐Sparing Robot‐Assisted Cystectomy With Intracorporeal Urinary Diversion be an Option?,” Case Reports 47 (2012): 72–75, 10.3109/21681805.2012.698317. 22746326

[13] Y. Jia , S. Li , and J. Liu , “Spontaneous Remission of Untreated Primary Amyloidosis of the Bladder After Transurethral Resection Biopsy: A Case Report and Literature Review,” Journal of International Medical Research 48 (2020): 300060520940452, 10.1177/0300060520940452. 33054497 PMC7580162

[14] D. G. Hof and L. Rasp , “Spontaneous Regression of Diffuse Tracheobronchial Amyloidosis,” Chest 76 (1979): 237–239, 10.1378/chest.76.2.237. 456066

[15] O. Costero , C. Riñón , F. Gil , et al., “Recurrence and Spontaneous Remission of Nephrotic Syndrome in Secondary Renal Amyloidosis,” Nefrología 22 (2002): 482–485. 12497751

[16] N. Guediri , I. Mejri , N. Boubaker , et al., “Spontaneous Resolution of a Pulmonary Cystic Amyloidosis Mass,” European Journal of Case Reports in Internal Medicine 9 (2022): 003586, 10.12890/2022_003586. 36506737 PMC9728224

